# Common Variants of *TLR1* Associate with Organ Dysfunction and Sustained Pro-Inflammatory Responses during Sepsis

**DOI:** 10.1371/journal.pone.0013759

**Published:** 2010-10-29

**Authors:** Maria Pino-Yanes, Almudena Corrales, Milena Casula, Jesús Blanco, Arturo Muriel, Elena Espinosa, Miguel García-Bello, Antoni Torres, Miguel Ferrer, Elizabeth Zavala, Jesús Villar, Carlos Flores, for the GRECIA and GEN-SEP groups

**Affiliations:** 1 CIBER de Enfermedades Respiratorias, Instituto de Salud Carlos III, Madrid, Spain; 2 Research Unit, Hospital Universitario N.S. de Candelaria, Tenerife, Spain; 3 Research Unit, Multidisciplinary Organ Dysfunction Evaluation Research Network, Hospital Universitario Dr. Negrin, Las Palmas de Gran Canaria, Spain; 4 Intensive Care Unit, Hospital Universitario Río Hortega, Valladolid, Spain; 5 Department of Anaesthesia, Hospital Universitario N.S. de Candelaria, Tenerife, Spain; 6 Pneumology Department, Clinic Institute of Thorax, Hospital Clinic, Barcelona, Spain; 7 Institut d'Investigacions Biomèdiques August Pi i Sunyer (IDIBAPS), University of Barcelona, Barcelona, Spain; 8 Surgical Intensive Care Unit, Department of Anesthesia, Hospital Clinic, Barcelona, Spain; 9 Keenan Research Center, Li Ka Shing Knowledge Institute, St. Michael's Hospital, Toronto, Canada; University of Giessen Lung Center, Germany

## Abstract

**Background:**

Toll-like receptors (TLRs) are critical components for host pathogen recognition and variants in genes participating in this response influence susceptibility to infections. Recently, *TLR1* gene polymorphisms have been found correlated with whole blood hyper-inflammatory responses to pathogen-associated molecules and associated with sepsis-associated multiorgan dysfunction and acute lung injury (ALI). We examined the association of common variants of *TLR1* gene with sepsis-derived complications in an independent study and with serum levels for four inflammatory biomarkers among septic patients.

**Methodology/Principal Findings:**

Seven tagging single nucleotide polymorphisms of the *TLR1* gene were genotyped in samples from a prospective multicenter case-only study of patients with severe sepsis admitted into a network of intensive care units followed for disease severity. Interleukin (IL)-1β, IL-6, IL-10, and C-reactive protein (CRP) serum levels were measured at study entry, at 48 h and at 7^th^ day. Alleles -7202G and 248Ser, and the 248Ser-602Ile haplotype were associated with circulatory dysfunction among severe septic patients (0.001≤*p*≤0.022), and with reduced IL-10 (0.012≤*p*≤0.047) and elevated CRP (0.011≤*p*≤0.036) serum levels during the first week of sepsis development. Additionally, the -7202GG genotype was found to be associated with hospital mortality (*p* = 0.017) and ALI (*p* = 0.050) in a combined analysis with European Americans, suggesting common risk effects among studies.

**Conclusions/Significance:**

These results partially replicate and extend previous findings, supporting that variants of *TLR1* gene are determinants of severe complications during sepsis.

## Introduction

Sepsis is a devastating clinical condition characterized by systemic inflammation occurring in the setting of a severe infection. Among intensive care unit (ICU) patients, incidence and mortality has been estimated in ∼12% and >40%, respectively, for the most severe forms [1]. Over the past three decades, a plethora of experimental and clinical studies have contributed substantially to our understanding of sepsis development and associated complications, including the acute lung injury (ALI) and the acute respiratory distress syndrome (ARDS) [2]. Studies in animal models, and twin and association studies have evidenced that the innate immune responses to pathogens show inter-individual variability that is strongly influenced by genetic factors, which may affect disease susceptibility and severity [3], [4], [5].

Toll-like receptor (TLR) pathways are critical components of the immune response to pathogens [6], and targeting of TLRs have been demonstrated to protect from lethal sepsis [7], [8]. Polymorphisms in genes of the TLR-mediated responses are associated with altered immunity [9], [10], [11], with susceptibility to infections and with related acute inflammatory syndromes, including sepsis and severe complications such as ALI and ARDS [12], [13]. In an elegant study, Wurfel and colleagues screened tagging single nucleotide polymorphisms (SNPs) across 49 TLR-related genes for association with whole blood inflammatory responses to pathogen-associated molecules in normal volunteers [14]. This allowed the identification of various tightly linked SNPs from the *TLR1* gene associated with the strongest hyper-inflammatory effects. In a subsequent association study in septic patients focusing on two of the common *TLR1* SNPs (-7202A/G and Ser602Ile) revealed associations with organ dysfunction, 28-day hospital mortality, ALI, and the prevalence of gram-positive cultures, constituting the unique association study of *TLR1* gene variants with sepsis-associated complications to date.

Given the controversies around the association of genetic factors with sepsis-associated outcomes because of the limited replicability of findings [15], [16], here we have examined the association of common variants of *TLR1* gene with sepsis outcomes in a cohort of patients with severe sepsis admitted into a network of ICUs from Spain, in order to provide an independent replication of previous results. We additionally explored the relationship of *TLR1* polymorphisms and of particular haplotypes with serum levels of four biomarkers of inflammation taken at three stages of sepsis development. We found that *TLR1* SNPs and haplotypes were associated with circulatory dysfunction and the source of infection among severe septic patients, and with hospital mortality and ALI in a combined analysis with data from European Americans. Congruently, risk alleles associated with increased nuclear factor κB (NF-κB) activation upon TLR1 stimulation in previous studies [17], [18], were related with reduced interleukin-10 and elevated C-reactive protein serum levels during the first week of sepsis development.

## Methods

### Ethics Statement

This study was approved by the Ethics Committees of Hospital Universitario N. S. de Candelaria and Hospital Universitario Río Hortega (Spain). Written informed consent was obtained from each subject or appropriate surrogates.

### Study Subjects

Samples were collected as part of a prospective, observational study of adults admitted during 2003–2005 into a network of Spanish ICUs. The study has been reported elsewhere [19] and included consecutive patients admitted to ICUs older than 18 years old fulfilling the international criteria for severe sepsis (n = 218) [20]. We collected basic demographic data, previous health status, severity of illness scores and clinical information until discharge from the ICU, including source of infection, pathogens, and development of organ dysfunction. All patients were followed prospectively for the development of ALI, as defined by the American-European Consensus Conference [21] and for the development of organ dysfunction included in the sequential organ failure assessment (SOFA) scale [22]. For the purpose of this study, patients with ALI and ARDS were analyzed as a single group of ALI patients. Genomic DNA was isolated from peripheral whole blood using commercial kits (GFX kit, GE Healthcare, Little Chalfont, UK) and stored at −20°C until use. A summary description of cases can be found in Table 1.

**Table 1:** Demographic and clinical features of patients with severe sepsis.

Variable		Cases (n = 218)
Gender, male (%)		59.2
Age, median years (P_25_–P_75_)		67 (54–75)
Previous health status (%)		
	Previous surgery	74.5
	Hospitalized >24 h	78.8
	Diabetes	16.1
	Hypertension	46.0
	Ischemic cardiac disease	9.3
	Smoker	27.6
Source of infection (%)		
	Pulmonary	34.4
	Extra-pulmonary	65.6
Identified pathogen (%)		
	Gram negative	23.9
	Gram positive	15.1
	Fungi	2.9
	Mixed Gram negative and positive	7.3
	Polymicrobial	2.0
	Negative blood cultures	48.8
Organ dysfunction (%)		
	Circulatory	83.1
	Neurologic	22.1
	Coagulation	22.1
	Renal	33.8
	Hepatic	15.5
APACHE II, median (P_25_–P_75_)		23 (18–27)
ALI or ARDS (%)		86.1
Hospital mortality (%)		52.7

Abbreviations: APACHE II, acute physiology and chronic health evaluation score II; ALI, acute lung injury; ARDS, acute respiratory distress syndrome.

DNA samples from population-based controls (n = 346), randomly drawn from a population-based study of about 7000 unrelated individuals as a representation of the general Spanish population [23], were also genotyped but data were only used for quality control purposes and not for association studies.

### SNP selection and genotyping

Instead of focusing exclusively on the two previously associated variants [14], we selected tagging SNPs (tSNPs) to efficiently study common *TLR1* gene variation in order to test whether additional gene variants associated with the expression of the clinical phenotype. TagIT 3.03 software [24] was used to select a set of 7 tSNPs from data available from 23 European-Americans retrieved from the Innate Immunity NHLBI Program for Genomic Applications (PGA) website (http://innateimmunity.net/IIPGA2/index_html, accessed October 2008), forcing the inclusion of previously associated tSNPs by Wurfel and colleagues [14]. A SNP-dropping-with-re-sampling method [24] was used to evaluate the expected properties of the *TLR1* 7 tSNPs set, indicating an average haplotype *r* ^2^>0.90.

Genotyping was conducted using the iPLEX™ Gold assay on MassARRAY system (Sequenom, San Diego, CA) by the Spanish National Genotyping Center, Santiago de Compostela Node (CeGen, http://www.cegen.org). Briefly, iPLEX™ assays were scanned by MALDI-TOF mass spectrometry and individual SNP genotype calls were automatically generated using Sequenom TYPER 3.4™ software. Two tSNPs that failed in iPLEX™ Gold assays (rs5743618 and rs5743551) were genotyped at Hospital Universitario N. S. de Candelaria using TaqMan™ assays (Applied Biosystems, Foster City, CA) in a 7500 Fast Real-Time PCR System (Applied Biosystems), with automated calls generated using the 7500 software 2.0.1 based on discriminating plots with 95% confidence. Genotyping was blind to sample outcomes. Approximately 10% of the samples were genotyped by duplicate to monitor genotyping quality. Genotypes were assigned using all data from the study simultaneously. Discrepancy rate among duplicates was 0.51% (95% confidence interval [CI]: 0.17–1.49%) for iPLEX™ Gold and 0.00% (95% CI: 0.00–2.11%) for TaqMan™. Call rates and further quality-control measures for genotyped tSNPs, as well as the correspondence of dbSNPs reference numbers with SNP positions relative to the start codon, can be found in Table 2.

**Table 2:** Quality control measures for the *TLR1* tSNPs.

rs#	tSNP position	Location	CR (%)	HWE *p*-value in controls
rs5743551	-7202A/G	5′ flanking	98.9	0.566
rs5743565	-5531A/G	Intron 1	99.1	0.294
rs5743594	-2299C/T	Intron 2	99.3	0.376
rs5743596	-2076C/T	Exon 3	99.3	0.469
rs5743611	238C/G (Arg80Thr)	Exon 4	98.8	1.000
rs4833095	742A/G (Asn248Ser)	Exon 4	99.3	1.000
rs5743618	1804G/T (Ser602Ile)	Exon 4	97.3	0.513

Abbreviations: CR, completion rate; HWE, Hardy-Weinberg equilibrium.

### Serum measurements

Serum was available only from a subset of 121 patients as dry-ice transportation to the coordinating center was not warranted for all participating centers. These samples were collected within 24 hours of meeting severe sepsis criteria, at 48 hours and 7 days after study entry, only if the patient remained hospitalized into the ICU. Given this, serum was available for all 121 patients at inclusion, for 96 of these patients at 48 hours, and for 60 of these patients at the 7^th^ day. Serum was obtained by centrifuging peripheral blood samples for 10 min at 3200 rpm within 35 min after sampling and was kept at −80°C until measurements were performed. These samples have been previously measured for four commonly used biomarkers of acute inflammation, interleukin (IL)-6, IL-1β, IL-10, and C-reactive protein (CRP) [25]. Measurements were done by duplicate using commercial kits: IL-6, IL-1β, and IL-10 were assessed in an Immulite analyzer (Siemens Medical Solutions Diagnostics, Caernarfon, UK), and CRP was determined in a Hitachi 917 analyzer (Roche Diagnostic, Basilea, Switzerland).

### Statistical analysis

Departures from Hardy-Weinberg equilibrium (HWE) were only tested in controls by means an efficient exact test [26]. Associations of SNPs with sepsis-derived complications were assessed using logistic regression models by means of SNPassoc [27] assuming additive effects for all SNPs. To replicate previous association findings [14], recessive models were also applied for -7202A/G and Ser602Ile SNPs as necessary. Logistic regressions were also used to obtain SNP effects as odds ratios (ORs) with 95% CI adjusting for age, gender, and the acute physiology and chronic health evaluation score (APACHE) II. Genotype data available from 20 unlinked polymorphisms from different parts of the genome [13] were utilized to adjust individual SNP associations for population stratification by means of a modified Cochran-Armitage trend test [28]. A Mantel-Haenszel test over genotype counts was used to perform the joint stratified analysis of the two SNPs overlapping across our study and the previous one [14], under the principle that if SNPs had consistent effects (i.e. allele and effect-wise) among different studies, the joint analysis would improve the results obtained from the two studies by separate. Data were analyzed under recessive models with -7202GG and 602Ile/Ile genotypes as risk factors to emulate previous association findings [14]. For this analysis, we did not consider non-septic control sample genotype counts from Wurfel et al. [14], and used data from the sepsis cohort to test associations with hospital mortality and the CELEG cohort to test associations with ALI development (Table S1). THESIAS [29] was used to assess the association of particular *TLR1* haplotypes with sepsis-derived outcomes. A two-tailed *p*≤0.05 was considered significant. However, in order to estimate the chance that SNP associations were true positives, we calculated the false-discovery rate (FDR) overall comparisons using QVALUE [30]. The patterns of linkage disequilibrium (LD), in terms of *r* ^2^ values, were explored using Haploview 3.32 [31].

Differences for serum biomarker levels between genotypes were explored using general linear models (GLM) for repeated measures in a longitudinal analysis over the three time points by means of SPSS 15.0 (SPSS Inc., Chicago, IL). Although the four biomarkers were measured for all 121 serum samples at inclusion, 96 serum samples at 48 h and 60 serum samples at 7^th^ day, the GLM analysis tested for biomarker level differences between genotypes over time. Thus, it only considered the 60 samples for which measures were available for the three time points. Despite dropping the sample size, GLM allowed to explore sustained SNP relationships with biomarkers, under the rationale that these were more likely to identify genuine associations. A *post hoc* analysis of differences in biomarker levels between genotypes at individual time points was also performed using all available serum measures from each day by means of linear regression. These analyses were performed under recessive models in order to emulate previous functional findings *in vitro* [17], [32], [33]. Values for IL-6, IL-1β, and IL-10 were logarithmically transformed for the analysis.

## Results and Discussion

Among the 218 severe septic patients, the median APACHE II score was 23 (interquartile range: 18–27), 55.0% percent developed ARDS and 31.1% developed ALI during hospitalization (Table 1). Peritonitis was the leading cause of severe sepsis followed by pneumonia, and most pathogens were characterized as Gram-negative bacteria. In agreement with previous studies [34], no pathogens were identified in blood cultures as the causative microorganism for sepsis albeit having an identified site of infection for 48.8% of patients. Hospital mortality was 52.7%.

Table 2 shows that completion rates were >97% for all 7 tSNPs and that all followed HWE expectations in control samples. None of the tSNPs was significantly associated with hospital mortality or with sepsis-induced ALI (Table 3). Borderline associations were observed for the SNP Arg80Thr (rs5743611) with hospital mortality (*p* = 0.080), and for SNPs -7202A/G (rs5743551) and Asn248Ser (rs4833095) with sepsis-induced ALI (*p* = 0.100 and *p* = 0.090, respectively). The association of -7202A/G with hospital mortality or with ALI did not improve if a recessive model was used. However, analyses combining samples from this and the previous study by Wurfel et al. [14], enabled the detection of a weak risk of the -7202GG genotype for hospital mortality and for ALI consistent across the two studies. The significance of the association of -7202A/G with hospital mortality improved to *p* = 0.017 (*p* = 0.028 in the previous study [14] and *p* = 0.566 in this study). Similarly, significance of the association of -7202A/G with ALI among septic patients improved to *p* = 0.050, while separate findings were non-significant (*p* = 0.171 in the previous study [14] and *p* = 0.108 in this study). For Ile602Ser (rs5743618), analyses combining samples from this and the previous study by Wurfel et al. [14], did not enable the detection of consistent significant effects (results not shown). Contrary to previous findings [14], none of the tSNPs showed a significant association with prevalence of Gram-positive infections versus other types of pathogens, but the SNPs -2299C/T (rs5743594) and -7202A/G showed borderline associations (*p* = 0.067 and *p* = 0.110, respectively). Nevertheless, in our study, three tSNPs had nominal significance in association with pulmonary infection among septic patients, which is frequently caused by Gram-positive bacteria [1], [35]: -5531A/G (rs5743565) with *p* = 0.004, -2076C/T (rs5743596) with *p* = 0.004, and Asn248Ser with *p* = 0.037. However, when adjusted for covariates using regression models, results remained significant for -5531A/G (OR for -5531A: 1.84, 95% CI: 1.10–3.10, *p* = 0.023) and for -2076C/T (OR for -2076C: 1.90, 95% CI: 1.07–3.39, *p* = 0.029), but not for Asn248Ser (OR for 248Ser: 1.44, 95% CI: 0.92–2.26, *p* = 0.112). Similar findings were obtained after applying a conservative correction for population stratification, as only the association of -5531A/G with pulmonary infection remained significant (*p* = 0.047, *p* = 0.057 and *p* = 0.127 for -5531A/G, -2076C/T and Asn248Ser, respectively).

**Table 3:** Association *p*-values of *TLR1* tSNPs with severe sepsis complications.

					Organ dysfunction				
tSNP	Hospital mortality	Acute lung injury	Gram positive infection	Pulmonary infection	Circulatory	Neurologic	Coagulation	Renal	Hepatic
-7202A/G	0.306 (0.566)	0.100 (0.108)	0.253 (0.110)	0.129 (0.399)	**0.001** (**0.008**)	0.621 (0.618)	0.464 (0.618)	0.641 (0.382)	0.657 (0.982)
-5531A/G	0.648	0.182	0.714	**0.004**	0.501	0.224	0.224	0.156	0.574
-2299C/T	0.818	0.845	0.067	0.803	0.111	0.935	0.935	0.428	0.737
-2076C/T	0.327	0.405	0.738	**0.004**	0.551	0.372	0.372	0.273	0.941
238C/G (Arg80Thr)	0.080	0.181	0.622	0.151	1.000	1.000	0.861	0.642	0.363
742A/G (Asn248Ser)	0.530	0.090	0.438	**0.037**	**0.009**	0.481	0.481	0.986	0.671
1804G/T (Ser602Ile)	0.164 (0.208)	0.142 (0.343)	0.129	0.309 (0.677)	**0.024**	0.557	0.310	0.988	0.463

In parenthesis, *p*-value for a recessive model; nominal significances in bold.

The strongest associations of *TLR1* tSNPs with sepsis-derived complications were found with circulatory dysfunction (Table 3). Three tSNPs, -7202A/G, Asn248Ser, and Ser602Ile, showed *p*-values ranging from 0.001 to 0.024. The association of -7202A/G remained significant after adjusting for covariates (OR for -7202G: 2.68, 95% CI: 1.40–5.12, *p* = 0.003) and population stratification (*p* = 0.031). The associations for Asn248Ser and Ser602Ile also persisted when adjusting for covariates (OR for 248Ser: 2.13, 95% CI: 1.16–3.90, *p* = 0.015; OR for 602Ile: 1.94, 95% CI: 1.10–3.44, *p* = 0.023) and showed borderline significance after the conservative adjustment for population stratification (*p* = 0.061 for Asn248Ser and *p* = 0.064 for Ser602Ile). In the context of the multiple tests performed, some of the above findings might represent true positives. In particular, FDR was below 5% for the association of -7202G/A with circulatory dysfunction, which partially replicates the study by Wurfel et al. [14], which reported the association of this *TLR1* SNP with circulatory, neurologic, coagulation, renal and hepatic dysfunction among septic patients. However, one must take into account the power limitation of this association study. Assuming a minor allele frequency of 30% (as is the case for -7202A/G) and an OR of 2.0, in the range of values reported by Wurfel et al [14], statistical power ranged from 39% to 69% for the outcomes tested, which might be responsible for the lack of replication in the association of TLR1 variants with some of the outcomes.

Many evidences point to the SNP Ser602Ile as the main responsible for the inter-individual variation in TLR1-mediated responses, 602Ser allele being related to a decrease in NF-κB activity [14], [17], [18], [32]. Albeit the SNP Asn248Ser have demonstrated no functional relevance [14], [18], previous association studies have indicated that it might be important for disease susceptibility [14], [36], [37], [38]. Thus, we were interested in testing the association of the ancestral haplotype 248Ser-602Ile [18] with sepsis-derived complications, motivated by the observation that this haplotype consistently tends to associate with the highest NF-κB activation upon TLR1 stimulation in independent studies [17]. In particular, we were interested in evaluating whether haplotypes of these two SNPs were more strongly associated with circulatory dysfunction than the two SNPs by separate. However, our results showed that, although 248Ser-602Ile haplotype homozygous subjects were more frequent among patients with circulatory dysfunction (17.5%) than in the rest of patients (5.6%), being a risk factor for circulatory dysfunction (OR: 2.07, 95% CI: 1.12–3.82, *p* = 0.020), statistical significance did not improve considerably compared to those obtained for Asn248Ser and Ser602Ile individually.

**Figure 1:**
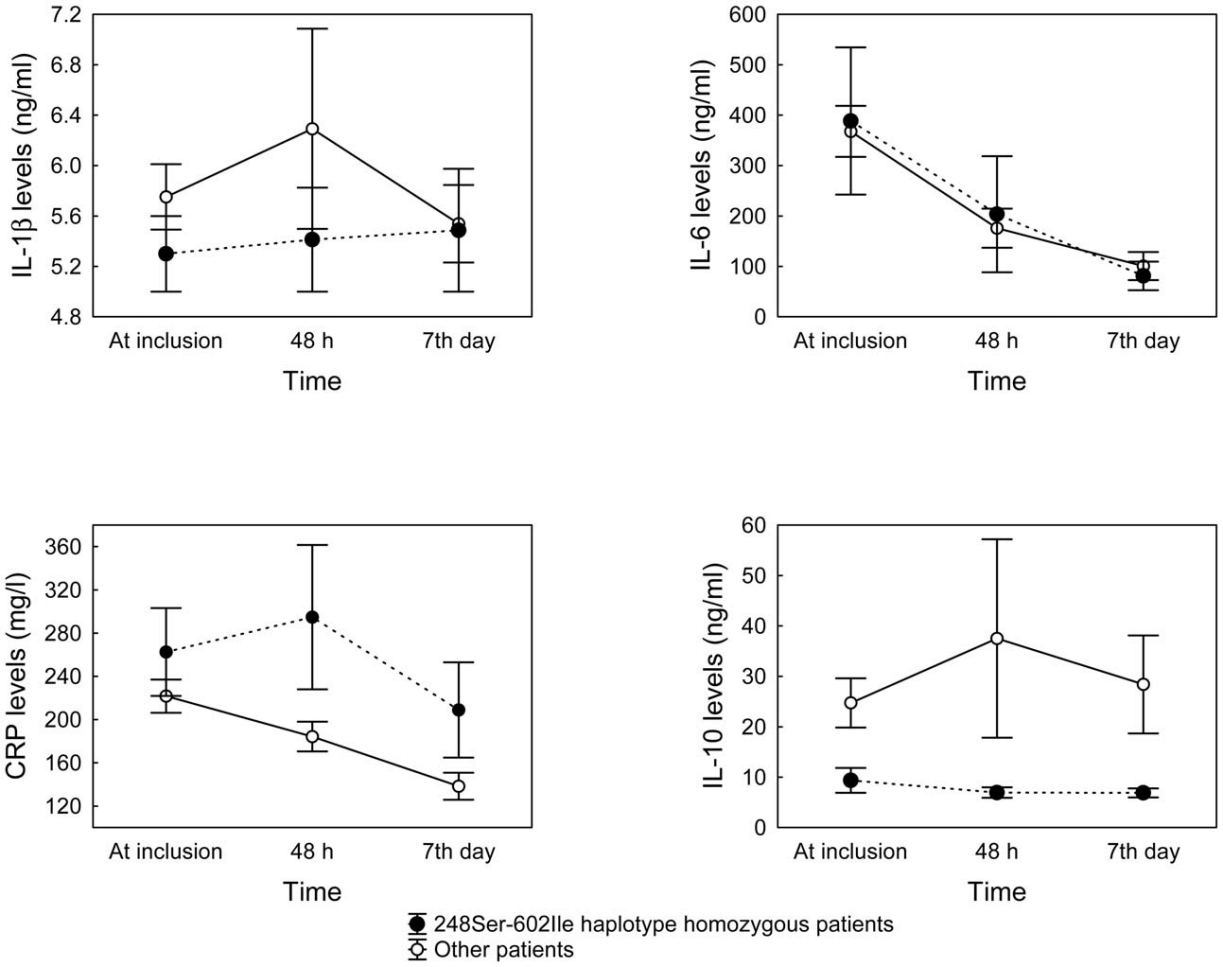
IL-6, IL-1β, IL-10, and CRP serum levels according to the *TLR1* 248Ser-602Ile haplotype status. Values represent mean ± SEM biomarker levels at inclusion, at 48 hours, and at 7^th^ day for patients showing homozygosity for the *TLR1* 248Ser-602Ile haplotype (n = 8) and for the rest of patients (n = 52). Note that values represented correspond to those from the 60 severe septic patients with serum measures available at the three time points. Significance of biomarker differences by the haplotype status was obtained using general linear models (GLM) for repeated measures in a longitudinal analysis assuming a recessive model (CRP, *p* = 0.024; IL-10, *p* = 0.015; for IL-1β and IL-6, *p*≥0.45).

A number of studies have explored the functional effects of *TLR1* variants *in vitro* [14], [17], [32]. However, no study have explored if *TLR1* variants are associated with amplified immune responses to pathogens *in vivo*. Given that serial serum measures for four biomarkers of inflammation were available from these patients during the first week after the onset of severe sepsis (Table S2), we correlated tSNPs genotypes with biomarker serial measures using GLM (Table 4). None of the tSNPs was found to be associated with serially-measured IL-1β and IL-6 serum levels, despite previous studies have demonstrated that *TLR1* gene polymorphisms determined IL-6 levels in response to specific gram-negative bacterial products [14]. On the other hand, CRP and IL-10 levels varied significantly among genotypes for four of the *TLR1* variants, with *p*-values ranging from 0.002 to 0.047. However, given the assessment used in this study to explore *TLR1* SNP relationships with biomarkers, statistical power was limited for most comparisons, particularly for those involving IL-6 and IL-1β for which it was below 30% (Table 4). Nevertheless, congruent with our previous results, homozygote patients for -7202G or 248Ser alleles, both constituting risk alleles for sepsis-associated complications, showed significantly increased levels for CRP (*p* = 0.036 and *p* = 0.012, respectively) and lower levels for the compensatory anti-inflammatory cytokine IL-10 (*p* = 0.011 and *p* = 0.047, respectively) (Table 4), regardless of the *in vitro* findings suggesting higher IL-10 levels for the -7202GG genotype in response to TLR1/TLR2 agonists [14]. This sustained pro-inflammatory state was also observed for patients homozygous for the risk haplotype 248Ser-602Ile (n = 8), associated with the highest NF-κB activation upon TLR1 stimulation [17], compared to the rest of patients (n = 52; *p* = 0.024 and *p* = 0.015 for CRP and IL-10 serial measures, respectively; *p*≥0.45 for IL-1β and IL-6) (Figure 1), slightly improving significance levels over individual SNP comparisons. Results for the longitudinal analysis barely changed when tests were performed under additive models or when adjusted for age, gender, and APACHE II (not shown).

**Table 4:** P-values and statistical power (%) from the longitudinal analysis of differences in serum biomarker levels by *TLR1* tSNPs.

tSNP	IL-1β		IL-6		CRP		IL-10	
	*p*-value	Power	*p*-value	Power	*p*-value	Power	*p*-value	Power
-7202A/G	0.714	6.5	0.277	19.0	**0.036**	56.1	**0.012**	72.2
-5531A/G	0.614	7.9	0.433	12.1	0.995	5.0	**0.009**	76.3
-2299C/T	0.527	9.6	0.408	13.0	0.288	18.4	0.413	12.8
-2076C/T	0.916	5.1	0.148	30.2	**0.002**	88.8	0.076	42.7
238C/G (Arg80Thr)	0.380	14.0	0.186	26.1	0.476	10.9	0.640	7.5
742A/G (Asn248Ser)	0.902	5.2	0.307	17.3	**0.011**	73.5	**0.047**	51.6
1804G/T (Ser602Ile)	0.685	6.9	0.320	16.7	0.129	32.8	0.117	34.7

Nominal significances in bold.

It is worth noting that no demographical or clinical differences were found between samples considered in the GLM analysis of *TLR1* SNP genotypes and serial measures of biomarkers, i.e. those patients who were not discharged from the ICU or were not dead before seven days, and patients without serum measures at the three time points, except for mortality (*p* = 0.033). Thus, it is reasonable to assume that this GLM biomarker analysis might be biased towards the expected for survivor patients. We explored such possibility by a *post hoc* analysis of differences in biomarker levels at individual time points among genotypes using all available serum measures. This analysis supported the strong correlation of *TLR1* SNPs with CRP and IL-10 levels observed before, while revealed barely significant relationships of *TLR1* SNPs with IL-6 and IL-1β levels (Table S3), indicating that GLM results were not as biased as predicted.

**Figure 2:**
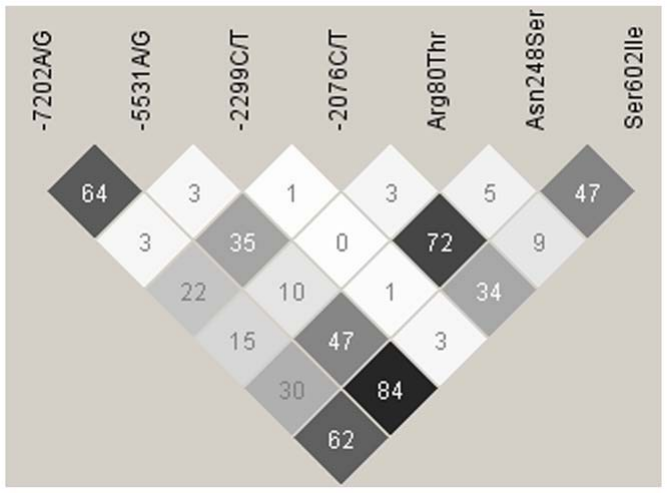
Linkage disequilibrium (LD) plot of *r* ^2^ values between *TLR1* SNPs. The plot was created with the genotype data from the Spanish population-based controls. Each diamond of the LD plot represents a pair-wise SNP comparison with its *r* ^2^ value indicated and schematically symbolized by a color gradient ranging from black (*r* ^2^ = 100, corresponding to complete LD) to grey (100>*r* ^2^>0, moderate LD) to white (*r* ^2^ = 0, corresponding to absence of LD).

Due to the remarkable effects on NF-κB activation, Ser602Ile has been put forward as the principal functional variant affecting *TLR1* activity across the whole chromosome region encompassing *TLR10*-*TLR1*-*TLR6* genes [18]. Nevertheless, we found stronger associations for SNPs -7202G/A and Asn248Ser with sepsis complications and with biomarker serum levels, in agreement with previous association studies [14], [36], albeit no functional properties have been described for them individually [14]. The LD between -7202G/A and Asn248Ser was strong (*r* ^2^ = 0.84), while the LD of these two with Ser602Ile was moderate in our sample (Figure 2), as has been found elsewhere [18], [39]. Furthermore, Asn248Ser have been associated with Leprosy and Malaria in populations where Ile602Ser is rare or absent [37], [38], indicating that it (or other variant in LD with it) might be relevant for susceptibility to infections. Given this, and the fact that associations found for Ser602Ile were weak unless considered into haplotypes, the evidences suggest that a more complex scenario, possibly involving other rare and common functional variants, underlie *TLR1* gene effect on the phenotype. Alternatively, unexplored epistatic interactions with other gene variants relevant for the TLR-mediated responses (e.g. *TLR4* Asp299Gly, TIRAP/Mal Ser180Leu, etc) might be needed to be able to detect Ser602Ile effects on this devastating clinical condition.

In conclusion, despite the limited power of the study, here we have replicated the association of *TLR1* gene variants, with established increased NF-κB activation *in vitro*, with risk for circulatory dysfunction during severe sepsis, and associated *TLR1* variants with elevated CRP and decreased IL-10 levels in serum during the first week of sepsis development. In line with previous evidences, our results suggest that genetic factors favouring an exacerbated host pro-inflammatory response to an infection predisposes to sepsis complications.

## Supporting Information

Table S1 Genotype counts for the *TLR1* SNPs used in meta-analysis.
(0.04 MB DOC)

Table S2 Mean (±SEM) serum levels of IL-6, IL-1β, IL-10 and CRP stratified by *TLR1* SNP genotypes.
(0.05 MB DOC)

Table S3 P-values from the *post hoc* analysis of differences in IL-1β, IL-6, CRP and IL-10 levels among *TLR1* genotypes at individual time points using all available serum measures from each time point (at inclusion, 48 hours, and 7^th^ day).
(0.04 MB DOC)
