# Supplementary material for: Common Variants of TLR1 Associate with Organ Dysfunction and Sustained Pro-Inflammatory Responses during Sepsis
Source: PLoS One. 2010 Oct 29;5(10):e13759. doi: 10.1371/journal.pone.0013759 (PMC2966434; doi:10.1371/journal.pone.0013759)
Supplement: Table S1 — Genotype counts for the TLR1 SNPs used in meta-analysis. (0.04 MB DOC) [file pone.0013759.s001.doc]

**Table S1. Genotype counts for the *TLR1*** SNPs used in meta-analysis.

| **SNP** | **Sample** | **Outcome** | **Genotype** | | |
| --- | --- | --- | --- | --- | --- |
| -7202A/G |  |  | A/A | A/G | G/G |
|  |  | Hospital mortality |  |  |  |
|  | Sepsis cohort^a^ | Death | 131 | 92 | 32 |
|  |  | Alive | 243 | 181 | 32 |
|  | This study | Death | 38 | 42 | 17 |
|  |  | Alive | 48 | 54 | 17 |
|  |  | ALI development |  |  |  |
|  | CELEG^a^ | ALI | 44 | 67 | 27 |
|  |  | Septic non-ALI | 41 | 47 | 13 |
|  | This study | ALI | 71 | 83 | 32 |
|  |  | Septic non-ALI | 15 | 13 | 2 |
| Ile602Ser |  |  | Ser/Ser | Ser/Ile | Ile/Ile |
|  |  | Hospital mortality |  |  |  |
|  | Sepsis cohort^a^ | Death | 116 | 87 | 40 |
|  |  | Alive | 217 | 166 | 61 |
|  | This study | Death | 29 | 42 | 24 |
|  |  | Alive | 38 | 53 | 25 |
|  |  | ALI development |  |  |  |
|  | CELEG^a^ | ALI | 54 | 62 | 11 |
|  |  | Septic non-ALI | 46 | 49 | 12 |
|  | This study | ALI | 54 | 83 | 44 |
|  |  | Septic non-ALI | 13 | 12 | 5 |

^a^From Wurfel et al. [14].
