# Supplementary material for: Common Variants of TLR1 Associate with Organ Dysfunction and Sustained Pro-Inflammatory Responses during Sepsis
Source: PLoS One. 2010 Oct 29;5(10):e13759. doi: 10.1371/journal.pone.0013759 (PMC2966434; doi:10.1371/journal.pone.0013759)
Supplement: Table S2 — Mean (±SEM) serum levels of IL-6, IL-1β, IL-10 and CRP stratified by TLR1 SNP genotypes. (0.05 MB DOC) [file pone.0013759.s002.doc]

**Table S2. Mean (± SEM) serum levels of IL-6, IL-1β, IL-10 and CRP stratified by *TLR1*** SNP genotypes.

| tSNP | Genotype (n) | IL-6 (ng/ml) | | | IL-1β (ng/ml) | | | IL-10 (ng/ml) | | | CRP (mg/l) | | |
| --- | --- | --- | --- | --- | --- | --- | --- | --- | --- | --- | --- | --- | --- |
|  |  | At inclusion | 48 h | 7^th^ day | At inclusion | 48 h | 7^th^ day | At inclusion | 48 h | 7^th^ day | At inclusion | 48 h | 7^th^ day |
| -7202A/G | G/G (8) | 500.1±130.0 | 202.6±101.2 | 87.8±67.1 | 5.3±0.6 | 5.4±1.9 | 5.5±0.8 | 10.5 ± 2. 5 | 6.2 ± 0.8 | 6.2 ± 0.6 | 258.5 ± 41.0 | 291.2 ± 68.2 | 203.9 ± 44.1 |
|  | A/A or A/G (52) | 350.7±51.0 | 176.0±39.7 | 99.7±26.3 | 5.8±0.3 | 6.3±0.8 | 5.5±0.3 | 24.6 ± 4.9 | 37.6 ± 19.7 | 28.5 ± 9.7 | 222.3 ± 15.4 | 184.8 ± 13.7 | 139.1 ± 12.6 |
| -5531A/G | A/A (38) | 397.7±59.9 | 215.0±45.8 | 121.6±30.4 | 5.7±0.3 | 6.6±0.9 | 5.6±0.3 | 24.9 ± 5.9 | 47.9 ± 26.8 | 34.7 ± 13.2 | 236.9±18.1 | 193.6±19.5 | 143.4±15.9 |
|  | G/G or G/A (21) | 323.8±78.8 | 118.3±60.2 | 57.5±39.9 | 5.6±0.4 | 5.4±1.1 | 5.4±0.5 | 18.4 ± 5.6 | 8.4 ± 0.9 | 9.6 ± 1.6 | 210.2±23.8 | 208.3±25.6 | 155.1±20.9 |
| -2299C/T | T/T (1) | 1000.0±361.8 | 66.3±286.1 | 130.0±189.9 | 5.9±1.7 | 5.0±5.4 | 5.0±2.1 | 79.5±32.2 | 9.9±133.5 | 12.2±66.1 | 311.0±111.7 | 303.0±119.4 | 238.0±97.3 |
|  | C/C or C/T (59) | 359.9±47.1 | 181.5±66.3 | 97.6±24.7 | 5.6±0.2 | 6.2±0.7 | 5.5±0.3 | 21.7±4.2 | 33.8±17.4 | 25.8±8.6 | 225.7±14.5 | 197.3±15.6 | 146.2±12.7 |
| -2076C/T | T/T (5) | 559.0±164.0 | 277.8±127.4 | 108.5±84.9 | 5.5±0.8 | 5.7±2.4 | 5.8±1.0 | 11.9±14.7 | 6.7±59.6 | 5.8±29.4 | 319.8 ± 42. 5 | 364.0 ± 94.1 | 226.4 ± 68.8 |
|  | C/C or C/T (55) | 353.5±49.5 | 170.6±38.4 | 97.2±25.6 | 5.7±0.2 | 6.2±0.7 | 5.5±0.3 | 23.6±4.5 | 35.9±18.0 | 27.3±8.9 | 218.7 ± 14.8 | 184.0 ± 13.1 | 140.5 ± 12.0 |
| Arg80Thr | Arg/Thr (6) | 385.8±149.0 | 60.2±116.7 | 44.1±77.7 | 6.7±0.7 | 6.1±2.2 | 5.9±0.9 | 21.0±13.6 | 14.0±54.9 | 27.3±27.2 | 218.7±46.2 | 134.0±48.5 | 150.0±40.0 |
|  | Arg/ Arg (53) | 357.0±50.1 | 194.1±39.3 | 105.7±26.2 | 5.6±0.2 | 6.2±0.7 | 5.5±0.3 | 23.0±4.6 | 36.1±18.5 | 25.7±9.2 | 227.5±11.5 | 207.7±16.3 | 149.2±13.5 |
| Asn248Ser | Ser/Ser (9) | 456.5±123.2 | 188.4±95.5 | 86.6±63.3 | 5.8±0.6 | 5.4±1.8 | 5.4±0.7 | 17.1 ± 8.1 | 7.3 ± 1.0 | 7.5 ± 1.0 | 267.9 ± 36.3 | 295.7 ± 58.9 | 212.1 ± 39.0 |
|  | Asn/Asn or Asn/Ser (51) | 355.4±51.7 | 178.0±40.1 | 100.1±26.6 | 5.7±0.2 | 6.3±0.8 | 5.5±0.3 | 23.6 ± 4.8 | 38.0 ± 20.1 | 28.7 ± 9.9 | 219.9 ± 15.6 | 181.9 ± 13.9 | 136.3 ± 12.6 |
| Ser602Ile | Ile/ Ile (12) | 388.4±106.3 | 226±84.2 | 159.0±55.5 | 5.3±0.5 | 5.3±1.6 | 5.8±0.6 | 13.1±9.7 | 8.2±39.3 | 45.9±19.3 | 251.9±32.7 | 253.9±34.6 | 177.2±28.6 |
|  | Ser/Ser or Ser/Ile (45) | 354.5±54.9 | 174.6±43.5 | 85.8±28.6 | 5.8±0.3 | 6.5±0.8 | 5.5±0.3 | 25.7±5.1 | 41.8±20.3 | 21.3±10.0 | 220.8±16.9 | 187.9±17.8 | 142.4±14.8 |
