# Supplementary material for: Common Variants of TLR1 Associate with Organ Dysfunction and Sustained Pro-Inflammatory Responses during Sepsis
Source: PLoS One. 2010 Oct 29;5(10):e13759. doi: 10.1371/journal.pone.0013759 (PMC2966434; doi:10.1371/journal.pone.0013759)
Supplement: Table S3 — P-values from the post hoc analysis of differences in IL-1β, IL-6, CRP and IL-10 levels among TLR1 genotypes at individual time points using all available serum measures from each time point (at inclusion, 48 hours, and 7th day). (0.04 MB DOC) [file pone.0013759.s003.doc]

**Table S3. P-values from the *post hoc* analysis of differences in IL-1β, IL-6, CRP and IL-10 levels among *TLR1* genotypes at individual time points using all available serum measures from each time point (at inclusion, 48 hours, and 7^th^ day).**

|  | IL-1β | | | IL-6 | | | CRP | | | IL-10 | | |
| --- | --- | --- | --- | --- | --- | --- | --- | --- | --- | --- | --- | --- |
|  | At inclusion  n=121 | 48 h  n=96 | 7^th^ day  n=60 | At inclusion  n=121 | 48 h  n=96 | 7^th^ day  n=60 | At inclusion  n=121 | 48 h  n=96 | 7^th^ day  n=60 | At inclusion  n=121 | 48 h  n=96 | 7^th^ day  n=60 |
| -7202A/G | 0.776 | 0.700 | 0.927 | **0.035** | 0.487 | 0.287 | 0.452 | **0.006** | 0.079 | 0.752 | 0.098 | 0.053 |
| -5531A/G | 0.095 | 0.345 | 0.870 | 0.779 | 0.199 | 0.518 | 0.706 | 0.858 | 0.659 | 0.618 | **0.039** | **0.045** |
| -2299C/T | **0.047** | 0.083 | 0.778 | 0.868 | **0.035** | 0.360 | 0.221 | 0.846 | 0.353 | 0.964 | **0.002** | 0.935 |
| -2076C/T | 0.855 | 0.845 | 0.787 | 0.093 | 0.120 | 0.898 | **0.024** | **<0.001** | 0.058 | 0.918 | 0.469 | 0.106 |
| 238C/G (Arg80Thr) | 0.409 | 0.770 | 0.561 | 0.927 | 0.128 | 0.572 | 0.871 | 0.217 | 0.985 | 0.199 | 0.777 | 0.405 |
| 742A/G (Asn248Ser) | 0.915 | 0.652 | 0.989 | 0.091 | 0.459 | 0.307 | 0.239 | **0.003** | **0.030** | 0.457 | 0.164 | 0.117 |
| 1804G/T (Ser602Ile) | 0.397 | 0.490 | 0.692 | 0.462 | 0.668 | 0.246 | 0.344 | **0.048** | 0.284 | 0.168 | 0.088 | 0.708 |

Nominally significant associations in bold.
